# Spread of competing viruses on heterogeneous networks

**DOI:** 10.1098/rsta.2016.0284

**Published:** 2017-05-15

**Authors:** Shanshan Chen, Kaihua Wang, Mengfeng Sun, Xinchu Fu

**Affiliations:** 1 Department of Mathematics, Shanghai University, Shanghai 200444, People’s Republic of China; 2 College of Mathematics and Statistics, Hainan Normal University, Haikou 571158, People’s Republic of China

**Keywords:** epidemic threshold, the basic reproduction number, pair-wise, competing viruses

## Abstract

In this paper, we propose a model where two strains compete with each other at the expense of common susceptible individuals on heterogeneous networks by using pair-wise approximation closed by the probability-generating function (PGF). All of the strains obey the susceptible–infected–recovered (SIR) mechanism. From a special perspective, we first study the dynamical behaviour of an SIR model closed by the PGF, and obtain the basic reproduction number via two methods. Then we build a model to study the spreading dynamics of competing viruses and discuss the conditions for the local stability of equilibria, which is different from the condition obtained by using the heterogeneous mean-field approach. Finally, we perform numerical simulations on Barabási–Albert networks to complement our theoretical research, and show some dynamical properties of the model with competing viruses.

This article is part of the themed issue ‘Mathematical methods in medicine: neuroscience, cardiology and pathology’.

## 1. Introduction

Despite centuries of efforts to improve public health and mitigate epidemic disease effects, the threat of infectious diseases remains. An important research method is epidemiology, with mathematical modelling as an analytical approach [1,2]. Mathematical modelling is an effective tool in understanding the spread of infectious diseases: it provides the best way of translating our knowledge about the status of disease transmission at the level of contacts between individuals to a description of its spread within the community [3–6].

However, most traditional models for describing disease propagation suppose that a population is uniformly mixing, and they overlook partnerships of contacts, so these models fail to describe epidemic propagation in large-scale social contact networks with distinct heterogeneity. For most diseases, epidemic spreading through physical interactions on contact networks can be used as the as practical grounds on which to model infectious disease dynamics [7–9]. The transmission of diseases on networks is based on the major connections between people who have a link with each other, that is to say, when considering the spread of an epidemic, it is the contact structure or the links between individuals that determine the progress of the disease within the population. Thus, the key idea is that one should consider the population of dyads, rather than just individuals [10]. Considering the ‘partnerships’ in contact on a network, pair approximation (PA) is a suitable method to depict it. Although the technique is not precise, it does capture some features of the spread. It takes into account pairs of connected individuals instead of just single individuals [11–13]. Research on pair-wise epidemic models in networks has received much attention since Matsuda applied PA to the research of population systems [14–19]. Many works have contributed to moment closure and PA, and these methods produce viable approximations to dynamics but are generally high dimensional and computationally intensive [20–25]. Volz and Miller solved this problem successively by applying the probability-generating function (PGF) to closure equations for a susceptible–infectious–recovered (SIR) model [26–30]. House and Keeling carried out further research into a new PGF moment-closure-based SIR model on heterogeneous clustered networks of heterogeneous links according to the Eames and Keeling model [25] and the Volz model [26,27], and then performed some numerical tests of the clustered PGF model, in contrast to simulation and other ordinary differential equation (ODE) approaches [31].

An important issue of interest in epidemiological research is the behaviour of competing pathogens. In many circumstances, a variety of strains with different infections, toxins and mobilities can exist together and compete for the same susceptible population. On the one hand, different strains can lead to different infectious states, and a conversion between different states may occur. On the other hand, one pathogen sometimes generates many strains with different spreading features. Competitive multi-virus propagation shows very rich behaviour, beyond that of single-virus propagation, hence a detailed investigation of multi-virus epidemic dynamics is required [32–35]. In fact, this occurs not only in the process of epidemic spreading; information transmission also shows competition with other information in the same population [36,37]. In [33], competing strains in heterogeneous networks were studied, and the existence of an epidemic threshold in the presence of other strains in the susceptible–infected–susceptible (SIS) model was confirmed by using the heterogeneous mean-field approach. In [38], the Miller–Volz model [28–30] was used to study two competing products within the same market. Their model shows that, if the two products have the same word-of-mouth marketing rate on the network, then the ratio of their market shares is exactly the same as the ratio of their advertisement rates. In these competitive spreading models, two or more viruses are exclusive: a node cannot be infected by two viruses simultaneously, that is to say, if a node (individual) is infected by one virus, it cannot be infected by the other one. This type of model has implications for many applications, such as product adoption (e.g. Apple versus Android smart phones), virus antidote propagation, opposing opinions about a subject, where people are for, against, or neutral, and so on [39]. But these methods cannot take the clustering coefficient into account, which is an important characteristic of network structure and has a significant impact on epidemic spreading [34,35,40–42].

The remainder of this paper is presented as follows. In §2, we begin with a review of the classical model with partnerships closed by PGF [31]. Then, we study the epidemic dynamics of this model under some assumptions via two methods, including the basic reproductive number and the stability of the model. In §3, we continue our discussion on the deterministic model described by House & Keeling [31], out of many different types of PAs, and propose our competing SIAR model, where I and A represent two competing viruses. At the same time, we derive the conditions for the local stability of this system. In §4, some numerical simulations are performed to complement the previous theoretical study. Numerical results show that some conditions in [33] are insufficient and that the clustering coefficient also affects the final state of infection.

## 2. The SIR model with the probability-generating function and its epidemic threshold

PA is the simplest kind of ‘moment closure mode’. It is closed on the tuple level, and gives a set of differential equations about tuple changes. In a static network, research on regular and random networks, whose degree of volatility is not too large, has been carried out previously [10–13]. In [26,27], Volz gave the pair-wise SIR model with the PGF and worked out the basic dynamical behaviour by some approximate methods. Then, Miller and Volz further simplified the Volz model [28–30]. However, this model did not involve ‘clustering’ or the heterogeneous degree, which are important characteristics of real contact networks. However, it provided an idea to reduce the dimensionality of the transmission models by using the pair-wise method.

### (a) The model with the probability-generating function

First, we revisit some results from the Volz model [27]. Volz defined a PGF 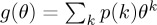 in an undirected random network with heterogeneous connectivity. Nodes can be in any of the three exclusive states: susceptible (*S*), infectious (*I*) or recovered (*R*). The dynamics are as follows: susceptible individuals may become infectious upon contact with infectious individuals, transmission causes new infection only along the edge of [*SI*] (or [*IS*]) with a constant rate *β* (i.e. *β* is a rate per edge along the edge). Infectious individuals become recovered at a constant rate *γ* after an infectious period and will never be infected again. All notation used is summarized in table 1.

**Table 1.** Notation used in models.

symbol	meaning
*N*,*N*_*k*_	the size of the network, the total fraction of nodes with degree *k* in the network
[*A*], [*A*_*k*_]	number of nodes in state *A*, number of nodes of type *A* with degree *k*
[*AB*]	number of pairs with one member in state *A*, and with the other member in state *B*
[*A*_*k*_*B*_*l*_]	number of pairs with one member in state *A* and with degree *k*, and with the other member in state *B* and with degree *l*
[*ABC*]	number of triples with one edge member in state *A*, with the middle member in state *B*, and with the other edge member in state *C*
[*A*_*k*_*B*_*l*_*C*_*m*_]	number of triples with one edge member in state *A* and with degree *k*, with the middle member in state *B* and with degree *l*, and with the other edge member in state *C* and with degree *m*
*C*_*AB*_	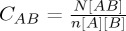 ; the correlation coefficient between states *A* and *B*
*p*_*k*_	proportion of nodes with degree *k*
*n*	the average degree of a vertex in the network ( 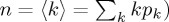
*Φ*	clustering coefficient of the network (equal to the number of triangles divided by the number of triples)
*θ*(*t*)	the fraction of degree 1 nodes that remain susceptible at time *t*
*Y*,*Q*	auxiliary variable used in the clustered PGF model ( 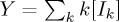 , 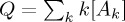 )
*g*(*x*)	PGF for the network degree distribution ( 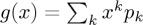 )

It is desirable to determine the dynamics of the number of susceptible and infected individuals and to develop equations in terms of these quantities. We note *S*(*t*), *I*(*t*) as the fraction of nodes susceptible, infected at time *t*, where *S*(*t*)=[*S*]/*N*, *I*=[*I*]/*N*. *S*_*k*_(*t*) is the fraction of nodes susceptible with degree *k* at time *t*, where *S*_*k*_=[*S*_*k*_]/*N*_*k*_. *P*_*I*_,*P*_S_ is the probability that an arc with a susceptible ego and an infectious, susceptible alter, where 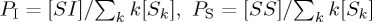.

A degree *k* susceptible node has an expected number *kP*_I_ of contacts with infectious nodes, and an expected number *βkP*_I_ d*t* of degree *k* susceptible node contacts will transmit disease to that node in a small time d*t*. It is clear that *S*_*k*_ is the fraction of degree *k* nodes in the network remaining susceptible at time *t*, and *S*′_*k*_=−*βkp*_I_(*t*)*S*_*k*_. We note *S*_1_=*θ*; therefore, the fraction of nodes that remain susceptible at time *t* is 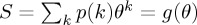, because 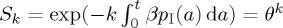. An edge in class *θ* loses its status only when it transmits, i.e. when a transmission occurs along it. The dynamics of *θ* are given by *θ*′(*t*)=−*βP*_I_(*t*)*θ*(*t*). The dynamics of *I*(*t*) are *I*′(*t*)=−*S*′(*t*)−*γI*(*t*).

Unfortunately, this does not form a closed system of differential equations, as both *θ*′(*t*) and *I*′(*t*) depend further on the dynamical variable *P*_I_. From the definition of *P*_I_, we have 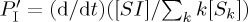. To obtain the derivative of [*SI*] and 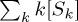, it requires careful consideration of the rearrangements of arcs from *S* to *S* and from *S* to *I*, as −*S*′ nodes become infected in a small time interval. Firstly, the derivative of 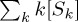 is easily placed in terms of *S*_*k*_ and *θ*.

To calculate [*SI*]′, Volz introduces the notation *δ*_*XY*_(*Z*) to represent the average excess *Z*-degree of nodes currently in disease state *X* selected by following a randomly chosen arc from node *Y* to *X* [27]. For example, *δ*_SI_(*I*) represents randomly choosing an edge from *I* to *S*, then following that arc to its destination (susceptible) node and finally counting all of the other edges emanating from that node to other infected nodes (ignoring the one along which it arrived). *δ*_SI_(*S*) give the average number of contacts to other susceptible nodes. The calculation of *δ*_*XY*_(*Z*) is straightforward and based on the current degree distribution of susceptible nodes in Volz [27].

Accordingly, [*SI*]′ includes the increasing or decreasing rate of [*SI*] at time *t*. Thence, [*SI*] is reduced at rate −*β*[*SI*], which accounts for all arcs which have an infectious ego which transmits to a susceptible alter; −*γ*[*SI*], which is due to the recovery of the *I* nodes; −*S*′*δ*_SI_(*I*)/*g*′(1), which represents *S* infected by its other infectious contacts by following that arc from one infectious node to this susceptible node. Similarly, [*SI*] is increased at rate −*S*′*δ*_*SI*_(*S*)/*g*′(1), which represents *S* infected by its other infectious contacts following that arc from one susceptible node to this susceptible node. Similarly, the dynamics for *P*_S_ can be derived analogously to the equation for *P*_I_.

The details of calculations are given in the Volz model [27] and result in the following model:
2.1
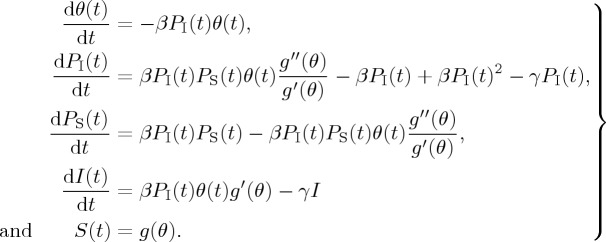
Assume that a small fraction of the nodes in the network are selected uniformly at random and initially infected, that is, *P*_S_(*t*)≈1, *θ*≈1, *P*_*I*_(*t*)≈0, [*I*(*t*)]≈0; Volz gave the epidemic threshold in terms of the transmissibility. We can obtained the corresponding basic reproductive number, which denotes the expected number of secondary infections caused by a single infected individual in a completely susceptible population. Accordingly, if *R*_0_<1, the disease will eventually disappear, and as long as *R*_0_>1 there will be a positive equilibrium representing an endemic status. The basic reproductive number of the Volz model is *R*_0_=(*β*/*γ*)(*g*′′(1)/*g*′(1)−1), where *g*′′(1)=〈*k*(*k*−1)〉,*g*′(1)=〈*k*〉=*n*. This is consistent with previous results based on bond-percolation theory [43].

Then, based on the above work and the results in [25–27], House and Keeling studied the full SIR pairwise equations of Eames & Keeling [25], and gave a ‘clustered PGF’ model using a relatively small number of ODEs [31], which is capable of capturing epidemic dynamics on clustered networks with a heterogeneous link distribution. The model consists of a system of six ODEs:
2.2
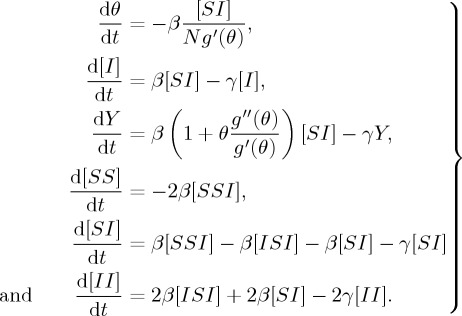
The above system is determined by the following model:
2.3
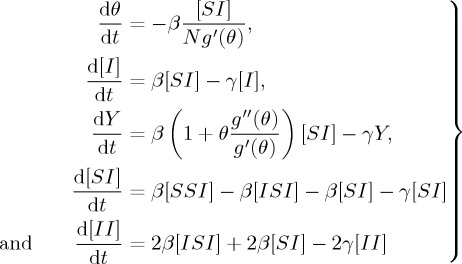
with [*SS*]+[*II*]+2[*SI*]=*nN*.

House and Keeling closed the two triples [*SSI*] and [*ISI*] that appear in the above unclosed pairwise SIR equations using the standard pairwise closure, the triple closure ([*A*_*k*_*B*_*l*_*C*_*m*_] ≈ ((*l* − 1)/*l*)[(1 − *Φ*)([*A*_*k*_*B*_*l*_][*B*_*l*_*C*_*m*_]/[*B*_*l*_]) + *Φ*(*nN*/*km*)([*A*_*k*_*B*_*l*_][*B*_*l*_*C*_*m*_][*C*_*m*_*A*_*k*_]/[*A*_*k*_][*B*_*l*_][*C*_*m*_])]), deconvolution of pairs ([*A*_*k*_*B*_*l*_]≈([*A*_*k*_*B*][*AB*_*l*_]/[*AB*])([*kl*]*nN*/*k*[*k*]*l*[*l*])), and the deconvolution of individuals ([*AB*_*k*_]≈[*AB*](*k*[*A*_*k*_]/*Σ*_*l*_*l*[*A*_*l*_])), so that the heterogeneity in both the link distribution and clustering can be analysed by using a low-dimensional model with a small number of dynamical variables. Then, the triples can be approximated by tuples and singles [31],

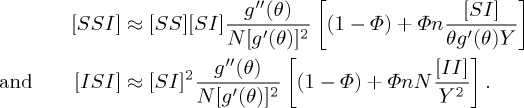


Although the dynamical model with respect to ‘closure (*Φ*)’ by the PGF method is low dimensional, there are few studies on its dynamical behaviour because of the complicated formula containing some variables, ‘Y, *θ*’, etc., in the denominators.

### (b) The stability of the disease-free equilibrium

In this section, we will undertake an analysis of the dynamical behaviour under some assumptions and approximations.

We first discuss the stability of equilibria for the model (2.2). Obviously, system (2.2) has zero equilibrium *E*_0_=(1,0,0,*nN*,0,0); we will use the Jacobian matrix near the equilibrium to derive the basic reproduction number of system (2.3).

**Figure 1.**
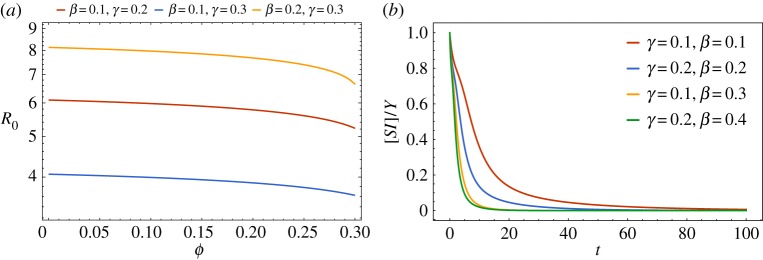
(*a*) *R*_0_ versus *Φ* of the SIR model closed by PGF; several sets of parameters are labelled using different colours; (*b*) [*SI*]/*Y* versus *t* under four sets of parameters *β* and *γ*. (Online version in colour.)

In the early spreading stage, if the population size is large enough, it is reasonable to assume that

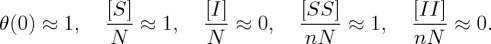
Furthermore, [*SI*]/*Y* is assumed to be equal to 1 initially, and the evolution of [*SI*]/*Y* with time is depicted in figure 1*b*. Therefore, this shows that the assumption of [*SI*]/*Y* ≈1 is reasonable in initial time.

Then, under the above basic assumptions, we obtain the approximation of some modules in the Jacobian matrix near *E*_0_. In 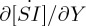, since *Y* ≈[*SI*], the module −*β*[*SS*][*SI*]^2^(*g*′′(*θ*)*Φn*/*N*[*g*′(*θ*)]^3^*θY* ^2^) can be approximated by −*βΦ*(*g*′′(1)/*g*′(1)) near *E*_0_.

Similarly, in 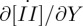, the module −4*β*[*II*][*SI*]^2^(*g*′′(*θ*)*Φn*/[*g*′(*θ*)]^2^*Y* ^3^) approximates to −4*β*(*g*′′(1)*Φn*/[*g*′(1)]^2^)([*II*][*SI*]^2^/*Y* ^3^); furthermore, [*II*][*SI*]^2^/*Y* ^3^ approximates to [*II*]/*Y* , which is close to [*II*]/[*SI*] and equivalent to *C*_II_[*I*]^2^/*C*_SI_[*S*][*I*]≈0 near *E*_0_.

Finally, we note that *g*′′(1)/*g*′(1)=*ξ*, and the corresponding Jacobian matrix near *E*_0_ is

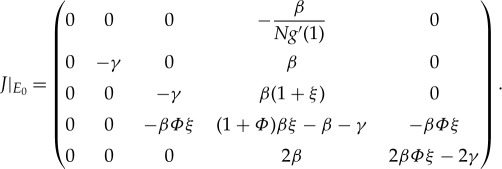


In order to analyse the eigenvalues of *J*|_*E*_0__, we give the characteristic equation:

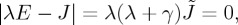
where

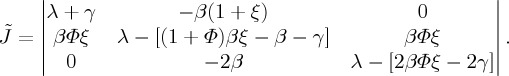
Then we only need to calculate the eigenvalues of 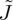; its characteristic equation is:
2.4
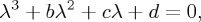
where *b*=4*γ*−3*βΦξ*−*βξ*+*β*, *c*=3*β*^2^*ξΦ*^2^+2*β*^2^*Φ*^2^*ξ*^2^−7*βΦξγ*−3*βξγ*+3*βγ*+5*γ*^2^+*β*^2^*Φξ*, *d*=4*β*^2^*Φξ*^2^*γ*+2*β*^2^*Φ*^2^*ξ*^2^*γ*−4*βΦξγ*^2^−2*βξγ*^2^+2*βγ*^2^+2*γ*^3^−2*β*^3^*Φ*^2^*ξ*^2^+2*β*^2^*Φξγ*−2*β*^3^*Φ*^2^*ξ*^3^.

By simply applying the ‘Sheng Jin formula’, which is the improvement of Cardano’s formula for solving the cubic equation [44], equation (2.4) has two conjugate imaginary roots and one real root. Then in order to judge the local stability of the disease-free equilibrium, we need to make sure that all eigenvalues of (2.4) have negative real parts. That is to say, the roots need to satisfy the following conditions:
2.5
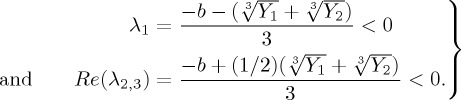
That is, 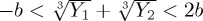, where 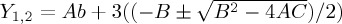, *A*=*b*^3^−3*c*, *B*=*bc*−9*d*, *C*=*c*^2^−3*bd*. From the above analysis, we have the following result.

**Theorem 2.1.**

*If* 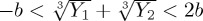
*, then the disease-free equilibrium E*_0_ *of the system (2.3) is locally stable; otherwise, it is unstable.*

Although the above method is convenient, it cannot derive the specific expression of the basic reproduction number. Let us consider the initial phase of an infection invading a total susceptible population. In line with requiring the infection states to increase in initial time, we can also study the issue according to the method presented in [45,46] under some conditions. Because [*S*] is assumed to be equal to *N* initially,

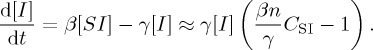

**Theorem 2.2.**

*Define R*_0_*=(βn/γ)C*_SI_*, when* 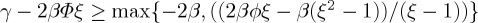
*. If R*_0_*<1, the disease-free equilibrium E*_0_ *of system (2.3) is locally asymptotically stable; otherwise, it is unstable.*

**Proof.**

*R*_0_=(*βn*/*γ*)*C*_SI_ relates to the parameters of the network structure average degree *n* and clustering coefficient *Φ*. We know that the correlation coefficient of state *C*_SI_ has a critical relationship with the basic reproduction number. So we consider the correlation coefficient of states *C*_*SI*_=*N*[*SI*]/*n*[*S*][*I*].

We also suppose that *θ*(0)≈1, [*S*]/*N*≈1, [*I*]/*N*≈0, [*SS*]/*nN*≈1, [*II*]/*nN*≈0. Then [*SI*]/*Y* ≈1 when *N* is large enough in the initial spreading. For system (2.3), we have

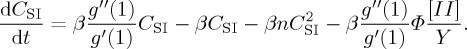
It is clear that the change rate also depends on the value of [*II*]/*Y* . It is of order 1, even when the density of infectious individuals is small [43]. So, by a similar method, we get d*C*_II_[*I*]/*N*/d*t*.

Then, if we denote *C*_SI_=*x*, [*II*]/*Y* =*y*, *g*′′(1)/*g*′(1)=*ξ*, we have
2.6
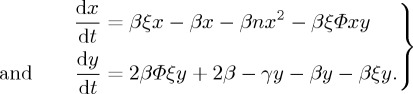
Now we discuss the existence and uniqueness of the positive solution of system (2.6).

Obviously, the positive equilibrium (x*,y*) of system (2.6) satisfies the following equations:

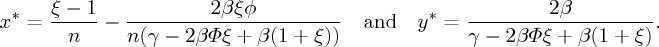

We get that, if *γ*−2*βΦξ*>(2*βϕξ*−*β*(*ξ*^2^−1))/(*ξ*−1), it can ensure that 0<x*,y*<1. Then, we consider *G*={(*x*,*y*) | 0<*x*,*y*<1}. Therefore, system (2.6) has the unique positive solution (x*,y*). Now it is necessary to prove the global stability of (x*,y*). We calculate the Jacobian matrix in the positive equilibrium (x*,y*) of (2.6),

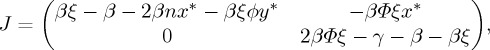
under the conditions of *γ*−2*βΦξ*>(2*βξ*−*β*(*ξ*^2^−1))/(*ξ*−1). We denote *a*_11_=*βξ*−*β*−2βnx*−βξϕy*, *a*_12_=−βΦξx*, *a*_21_=0, *a*_22_=2*βΦξ*−*γ*−*β*−*βξ*.

Clearly,

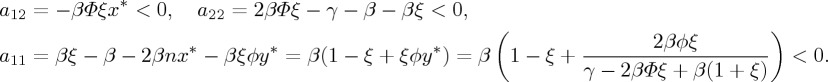
In that way, all real parts of the eigenvalues of the Jacobian matrix are negative. Hence, the positive equilibrium (x*,y*) is locally asymptotically stable. Similarly, we find that (0,y*) is unstable.

Now we further discuss the global stability of the positive equilibrium (x*,y*). For system (2.6), in *G*, we denote *P*(*x*,*y*)=*βξx*−*βx*−*βnx*^2^−*βξΦxy*, *Q*(*x*,*y*)=2*βΦξy*+2*β*−*γy*−*βy*−*βξy*. Let *B*(*x*,*y*)=1, then *D*=∂(*BP*)/∂*x*+∂(*BQ*)/∂*y*=(−2*βnx*−*βξy*−2*β*+2*βΦξ*−*γ*), so the condition *γ*−2*βΦξ*≥−2*β* should be ensured for the sake of the fixed sign of *D*, and, also, it is not identically equal to zero in any subregion. Therefore, based on the ‘Bendixson–Dulac criteria’ [47,48], when *γ*−2*βΦξ*≥−2*β* and *γ*−2*βΦξ*>((2*βξ*−*β*(*ξ*^2^−1))/(*ξ*−1)), system (2.6) does not have limit cycles in *G*. So, the positive equilibrium point is globally stable. So, we obtain *R*_0_=βnx*/*γ*. ▪

## 3. The SIAR model with the probability-generating function and its epidemic threshold

The process of one epidemic spreading usually prevents many other viruses spreading, or one pathogen sometimes generates many strains with different spreading features [32,49,50]. A problem of some interest in multi-strain spreading is the behaviour of competing viruses. They will compete with populations with different infectious rates and final states.

In this section, we will construct an SIAR model depicting two kinds of viruses from the same pathogen or two distinct pathogens competing with each other at the expense of common susceptible individuals by using the pair-wise method and closed by PGF. The two kinds of viruses are named *I* and *A*. In our competitive spreading model, the two viruses are exclusive: a node cannot be infected by virus *I* and virus *A* simultaneously, and the ‘virus’ may refer to pathogens, products or two opposite views. Let the spreading rate of *I* be *β* and that of strain *A* be *μ*; the recovery rates are *α*_1_ and *α*_2_, respectively. On a network, every node represents an individual, then the edges are the links between the individuals. Each individual can be in one of the three states: susceptible to the disease, infectious when they can spread the disease to the susceptible, and recovered when they have been infected but can no longer spread or catch the disease. However, the equations describe the behaviour of [*AB*] pairs, instead of just the behaviour of individuals, which is different from [33]. Out of many different types of PAs, we follow in our discussion the deterministic model described by House & Keeling in [31] and some studies in §2.

### (a) The SIAR model

According to the discussion in §2, our two-virus model with a competing mechanism using the moment closure PGF can be described and determined as follows:
3.1
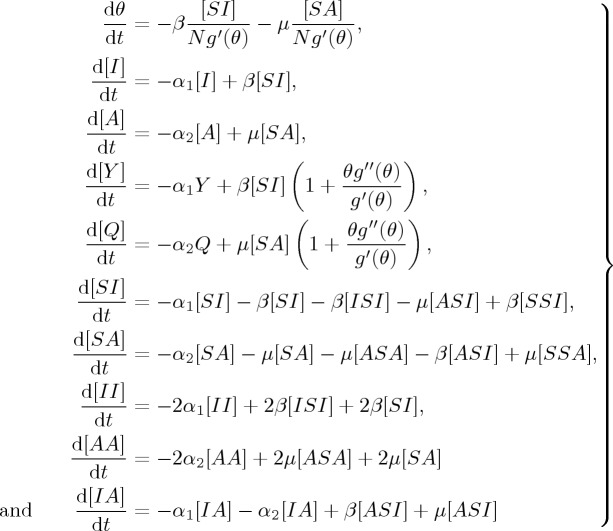
with [*SS*]+[*II*]+[*AA*]+2[*SI*]+2[*SA*]+2[*IA*]=*nN*. The triples will be approximated by tuples and singles, which can capture epidemic dynamics on clustered networks with a heterogeneous link distribution:

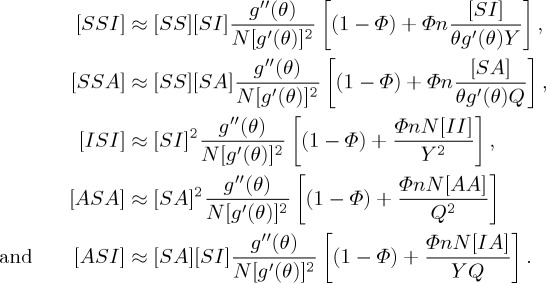


### (b) The basic reproduction number

In the following, we will derive the basic reproduction number for system (3.1) from the eigenvalues of the Jacobian matrix at disease-free equilibrium. Obviously, system (3.1) has the disease-free equilibrium *E*_0_, where [*I*]=[*A*]=[*AA*]=[*II*]=[*IA*]=[*SA*]=[*SI*]=*Y* =*Q*≈0, *θ*≈1, [*S*]≈*N*, [*SS*]≈*nN*. A similar discussion has been given in §2 for the SIR model by PGF. In the early stage of the spreading of the virus, we may assume that [*SI*]/*Y* ≈1. Therefore, the corresponding matrix of system (3.1) near *E*_0_ is

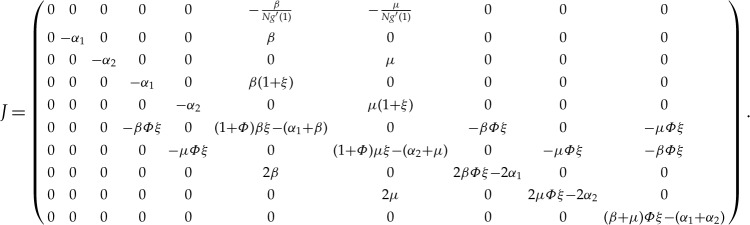
Now we discuss the eigenvalues of the above matrix in the following two cases.

**Case 3.1.**

Consider the special case first: the clustering coefficient *Φ*=0. In this case,

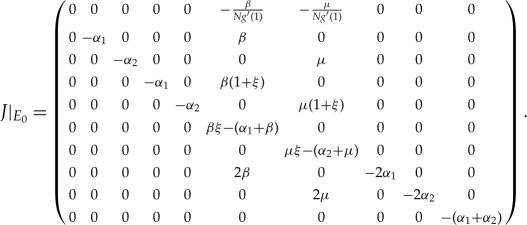
The eigenvalues corresponding to this Jacobian satisfy the following identity:

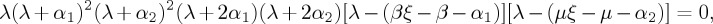
which implies that, in order to ensure the stability of *E*_0_, if and only if *βξ*−*β*−*α*_1_<0 and *μξ*−*μ*−*α*_2_<0, we note *R*_01_=*β*(*ξ*−1)/*α*_1_ and *R*_02_=*β*(*ξ*−1)/*α*_1_; that is, *R*_01_<1, *R*_02_<1. According to the results in [27], we know that they are the basic reproduction numbers of the SIR and SAR models, respectively, when there is only one virus spreading in the network. Therefore, we derive the basic reproduction number 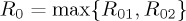. Obviously, the disease-free equilibrium *E*_0_ is stable in the SIAR model if and only if the disease-free equilibria are stable in both the SIR and SAR models.

**Case 3.2.**

The general case: *Φ*≠0. Let *R*_01_ and *R*_02_ represent the basic reproduction numbers of the SIR and SAR models, respectively. The characteristic equation |*λE*−*J*|=0 leads to

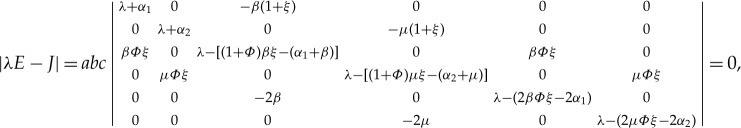
where we denote *λ*(*λ*+*α*_1_), (*λ*+*α*_2_) and *λ*−[(*β*+*μ*)*Φξ*−(*α*_1_+*α*_2_)] as *a*, *b* and *c*, respectively, and

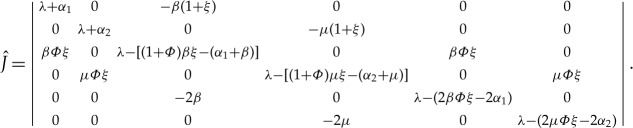

Through some similar transformation of the matrix 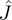, it is simplified to the following form:

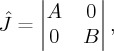
where

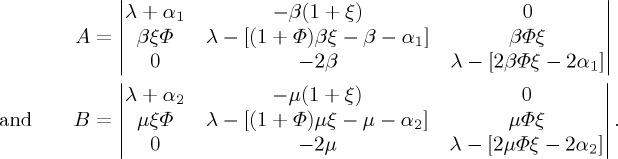
Obviously, according to the discussions in §2, matrices *A* and *B* correspond to the characteristic equations of the Jacobian matrices of the SIR and SAR models near their disease-free equilibria *E*_0_, respectively. Therefore, in the SIAR model, the characteristic equation of the Jacobian matrix near *E*_0_ satisfies the following equation:

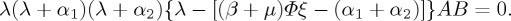
Therefore, the disease-free equilibrium *E*_0_ is locally stable if and only if all of the eigenvalues are negative, equivalently,
3.2
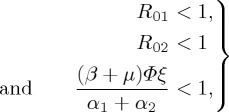
where *R*_01_ and *R*_02_ are the basic reproduction numbers of the SIR and SAR models, respectively, which are the expected numbers of secondary infections caused by a single-infected individual in a completely susceptible population if there is only one virus spreading in the network. The expression of (*β*+*μ*)*Φξ*/(*α*_1_+*α*_2_) shows that *R*_0_ of the SIAR model also depends on the clustering coefficient *Φ* during the period of the transmission of virus *A* and *I*. Then, in the SIAR model, as long as we require the basic reproduction number

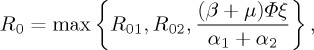
we can obtain the following result.

**Theorem 3.3.**

*Define* 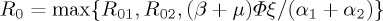
*, if R*_0_*<1, then the disease-free equilibrium E*_0_ *of system (3.1) is locally stable; otherwise, it is unstable.*

This shows clearly that, in the two-strain competing model, only controlling their own spreading thresholds is not enough. The value of (*β*+*μ*)*Φξ*/(*α*_1_+*α*_2_) also has a vital influence on the state of infection, that is to say, the spreading threshold of the two-strain competing model not only depends on the degree distribution, but also is related to the clustering coefficient of the network.

## 4. Numerical simulations and analysis

Because of the complicated nature of the SIAR model, it is difficult to analyse its global dynamics by a rigorous theoretical method. So in this section, we will provide further analysis of the transmission dynamics by numerical simulations.

We perform the simulations on networks not only to verify our theoretical results obtained in the previous sections, but also to reveal some new phenomena which are difficult to obtain by theoretical means. Our simulations are based on the Barabási–Albert network, with network size *N*=500, degree distribution *p*(*k*)∝*k*^−2.57^, average degree *n*=5.976, and *Φ*=0.0657.

From the analysis of the basic reproduction number of the model (2.3) in §2b, *R*_0_=*βn*/γx*, which depends on the clustering coefficient *Φ*. Figure 1*a* shows the effect of the clustering coefficient *Φ* on *R*_0_. It is clear that, with increasing *Φ*, *R*_0_ is decreasing under the condition that the degree distribution remains unchanged, which is the same as the conclusion in [34] for the model with random and regular contacts by the pair-wise method.

**Figure 2.**
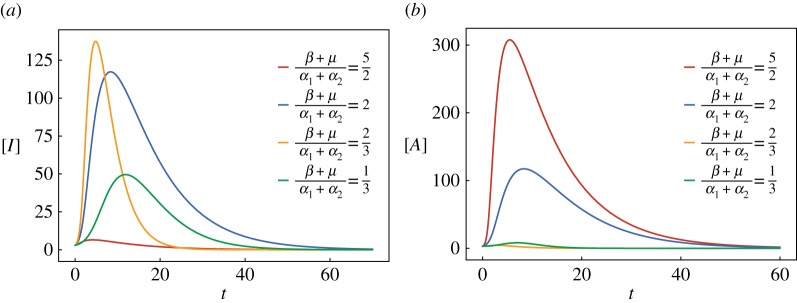
(*a*) The effect of parameters with infectious size of [*I*] and (*b*) the effect of parameters with infectious size of [*A*]. (Online version in colour.)

As shown in figure 2, we consider how the value of ((*β*+*μ*)/(*α*_1_+*α*_2_)) influences the epidemic spreading of SIAR model in §3 with four sets of parameters:

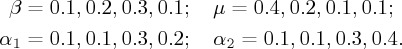
It is clear that the final state is critical to the relationship between *β* and *μ*. As long as *β*<*μ* (*β*>*μ*) and (*β*+*μ*)/(*α*_1_+*α*_2_)>1, the virus [A] ([I]) can defeat [*I*] ([*A*]) occupying the network, which corresponds to the solution (0,A*) ((I*,0)) to system (2.8). However, if (*β*+*μ*)/(*α*_1_+*α*_2_)<1, the condition is the same, but the infection scale will decrease. Then, paying attention to the second and third sets of parameters, when *β*=*μ*, no matter whether (*β*+*μ*)/(*α*_1_+*α*_2_)>1 or <1, the two competing viruses will coexist. However, it is noteworthy that when (*β*+*μ*)/(*α*_1_+*α*_2_)<1 the infection scale is lower than the case of (*β*+*μ*)/(*α*_1_+*α*_2_)>1.

**Figure 3.**
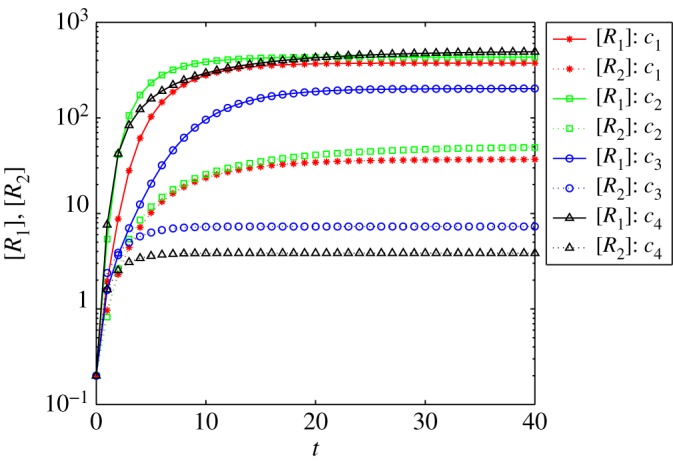
The sizes of *R*_1_ and *R*_2_ versus *t* in logarithmic coordinates. Solid line: the size of *R*_1_; dashed line: the size of *R*_2_. Where under the following cases: the red line with stars *C*_1_ satisfies *R*_01_>1, *R*_02_>1,*R*_03_<1; the green line with squares *C*_2_ satisfies *R*_01_>1, *R*_02_>1,*R*_03_>1; the blue line with circles *C*_3_ satisfies *R*_01_>1, *R*_02_< 1,*R*_03_<1; and the black line with triangles *C*_4_ satisfies *R*_01_>1, *R*_02_<1,*R*_03_>1. (Online version in colour.)

Figure 3 depicts the relationship between the ultimate sizes of *R*_1_ and *R*_2_ for the competing diseases *I* and *A* under different cases in logarithmic coordinates. We note that, according to §2, 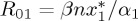, 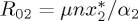, and *R*_03_=(*β*+*μ*)*Φξ*/(*α*_1_+*α*_2_). The lines *C*_1_,*C*_2_ show that two competing viruses will lead to large-scale outbreaks under the conditions *R*_01_>1,*R*_02_>1,*R*_03_<1, *R*_01_>1,*R*_02_>1,*R*_03_>1. From *C*_3_ and *C*_4_, we can see that only *I*(*t*) has large-scale outbreaks on the network when *R*_01_>1,*R*_02_<1. While we see similar phenomena in figure 2, the outbreak scale is lower when *R*_03_<1 than when *R*_03_>1. Therefore, from figures 2 and 3, we see that, as long as there is a value *R*_01_ or *R*_02_ greater than 1, disease will be prevalent on the network; whereas if *R*_03_>1, disease will have a massive outbreak on the network.

## 5. Conclusion

When we consider the spread of an epidemic, it is the contact structure between individuals that determines the progress of the disease through the population. Correlation models, and, in particular, pair-wise models, have been primarily used to describe the behaviour of simple spatial models. In addition, generally speaking, the forms of interaction between two epidemic particles in a multi-strain epidemic model may contain many types, but the competing strains are relatively simple. However, because of the complicated nature of the approximated forms by pair-wise modelling, the further study of epidemic dynamics with this model is limited using the present methods.

Now we give a brief summary of this paper. We have derived the basic reproduction number of the basic SIR model closed by PGF. Then, we proposed an SIAR model on heterogeneous networks with pair-wise modelling closed by PGF. By theoretical analysis and numerical simulation, we described the effect of a pair-wise model on the spreading dynamics of two competing viruses, and found the conditions to ensure the local stability of a disease-free equilibrium. The stable condition not only is related to the basic reproduction number of SIR and SAR models, but also connects with (*β*+*μ*)*Φξ*/(*α*_1_+*α*_2_).

In this paper, we have only studied the simplest case of competing pathogens. The concept of competition between the two strains of infection (or pathogens) means that two strains from one pathogen cannot co-infect in a single host at the same time. There are also other forms of interaction between two pathogens except competing virus, such as super-infection, which means that one more virulent pathogen can outcompete the other less virulent pathogen; or co-infection, which means that two pathogens can be hosted in one individual. These processed may also be modelled by using pair-wise modelling closed by PGF, which will be further analysed in our future research.
